# A Multidomain Approach to Assessing the Convergent and Concurrent Validity of a Mobile Application When Compared to Conventional Methods of Determining Body Composition

**DOI:** 10.3390/s20216165

**Published:** 2020-10-29

**Authors:** Eric V. Neufeld, Ryan A. Seltzer, Tasnim Sazzad, Brett A. Dolezal

**Affiliations:** 1 Airway & Exercise Physiology Research Laboratory, David Geffen School of Medicine, Los Angeles, CA 90095, USA; eneufeld8@ucla.edu (E.V.N.); sazzad.tasnim@gmail.com (T.S.); bdolezal@mednet.ucla.edu (B.A.D.); 2 Donald and Barbara Zucker School of Medicine at Hofstra/Northwell, Hofstra University, Hempstead, NY 11549, USA; 3 School of Medicine, Stanford University, Stanford, CA 94305, USA

**Keywords:** anthropometry, digital health, waist-to-hip ratio, body composition, body fat percentage, validity, health monitoring

## Abstract

Determining body composition via mobile application may circumvent limitations of conventional methods. However, the accuracy of many technologies remains unknown. This investigation assessed the convergent and concurrent validity of a mobile application (LS) that employs 2-dimensional digital photography (LS2D) and 3-dimensional photonic scanning (LS3D). Measures of body composition including circumferences, waist-to-hip ratio (WHR), and body fat percentage (BF%) were obtained from 240 healthy adults using LS and a diverse set of conventional methods—Gulick tape, bioelectrical impedance analysis (BIA), and skinfolds. Convergent validity was consistently high—indicating these methods vary proportionally and can thus reliably detect changes despite individual measurement differences. The span of the Limits of Agreement (LoA) using LS were comparable to the LoA between conventional methods. LS3D exhibited high agreement relative to Gulick tape in the measurement of WHR, despite poor agreement with individual waist and hip circumferences. In BF%, LS2D exhibited high agreement with BIA and skinfold methods, whereas LS3D demonstrated low agreement. Interestingly, the low inferred bias between LS3D and DXA using existing data suggests that LS3D may have high agreement with dual-energy x-ray absorptiometry. Overall, the suitability of LS2D and LS3D to replace conventional methods must be based on an individual user’s criteria.

## 1. Introduction

Lifestyle-related metabolic diseases constitute some of the most pervasive yet preventable ailments in the developed world. An underlying symptom that substantially increases the risk for developing these conditions is excess body weight and obesity [1,2,3]. Although many pharmacological, surgical, and behavioral interventions exist to combat obesity, it remains a prominent and expanding public health issue [4]. Effectively combatting obesity requires a multidisciplinary and personalized approach, which is increasingly intertwined with the rapidly advancing field of digital health technology.

Mobile applications and wearable devices are increasingly used to track a wide variety of physiological parameters. Many of these are aimed at prevention and mitigation of metabolic disease—such as using heart rate and energy expenditure to promote physical activity and healthy dietary intake [5,6,7,8,9]. This trend is in line with the current vast expansion in use of digital health technology for non-contact medical evaluation during the COVID-19 pandemic [10,11,12]. However, not all digital health technology utilizes scientifically established guidelines and, those that do may still lack accuracy or precision [13,14,15,16]. Technological advancement naturally outpaces validating rigorous research, thus the quality of many health-related mobile applications remains unknown.

Monitoring various measurements of body composition can promote a reduction in obesity and decreased risk of metabolic disease; however, many conventional measurements are often expensive, cumbersome, or best measured by a trained provider. Self-weighing on a consistent basis, arguably the simplest method of quantifying body composition, improves weight outcomes without adverse psychological effects [17,18]. Although this can be easily converted into a body mass index (BMI), this method has limited clinical utility due to its poor sensitivity and overall simplicity [19,20,21]. In addition to body weight, body composition measures closely linked to metabolic diseases include waist circumference, waist-to-hip ratio (WHR), and body fat percentage (BF%) [22,23,24]. Although helpful in providing a fuller picture of body habitus and characterizing disease risk, many of these require substantial training, have limited efficacy in certain populations, and may be prohibitively expensive. For example, measuring tapes can be simplistic and inefficient, dual-energy x-ray absorptiometry (DXA) is expensive and often inaccessible, and bioelectrical impedance analysis (BIA) can exhibit poor accuracy in certain populations while requiring stringent pretesting standards [25].

Digital anthropometry via mobile application has the potential to determine body composition while circumventing several limitations of conventional techniques. LeanScreen (LeanScreen^®^, version 8.6; PostureCo, Inc., Trinity, FL, USA) is a mobile application that provides a cluster of body composition measures through novel, non-contact 2D digital photography (LS2D) and 3D photonic scanning (LS3D). While existing evidence suggests these 2D [26] and 3D technologies are valid measurement tools, recent studies have called the accuracy and utility of LeanScreen (LS) into question [27]. Specifically, Macdonald et al. found a consistent and significant underestimation of BF% as determined by LS2D technology (version 6.0) compared to DXA [27]. For LS2D, they reported a combined male-female (n = 148) bias of −3.3 ± 3.6 with limits of agreement (LoA) spanning 13.99 (−10.26 to 3.73). However, they also concluded that LS2D exhibits high inter-rater and intra-rater reliability.

The objective of this investigation was to evaluate both the convergent and concurrent validity of LS2D and LS3D technology in determining body composition (body circumferences, WHR, and BF%) when compared to a diverse set of conventional methods (Gulick tape, BIA, and skinfolds). This breadth of direct comparison provides a higher degree of confidence and transparency for individuals seeking to compare LeanScreen against their preferred method of determining body composition.

## 2. Methods

### 2.1. Participants

Our sample consisted of 153 men and 87 women (mean ± SD: age: 20.8 ± 2.3 years; height: 172.3 ± 9.8 cm; weight: 69.5 ± 12.2 kg; BMI: 23.3 ± 2.8 kg/m^2^). The majority were visibly healthy undergraduate students that engaged in routine moderate-to-vigorous exercise (7.0 ± 3.9 h/week). Participants comprised a multitude of races including Caucasian (n = 113), African American (n = 6), Asian (n = 80), and non-white Hispanic (n = 25). Some individuals reported mixed descent such as Caucasian/Asian (n = 11) and Caucasian/Hispanic (n = 5).

Participants 18–40 years old were recruited using flyers posted on the UCLA campus and by email. Exclusion criteria included: (i) pregnancy, (ii) metal or silicone body implants, and (iii) cancer diagnosis or receiving radiological treatment. Each participant reported to the UCLA Airways & Exercise Physiology Research Laboratory for a single 30-min session. During this session, three body composition measures—Body Circumference, Waist-to-Hip Ratio (WHR), and Body Fat Percentage (BF%)—were collected using conventional (criterion) methods and LeanScreen to enable calculation of concurrent and convergent validity (Table 1). The UCLA Institutional Review Board approved this study (IRB#11-003190), and all participants provided informed consent prior to enrollment.

### 2.2. Measurements with Conventional Methods and LeanScreen

Each measure of body composition—body circumferences, Wait-To-Hip Ratio, and BF%—was collected using conventional methods and LeanScreen photographic or 3D-scanning methods (Table 1). All measurements for a participant were collected in a single session while the participant wore minimal clothing—fitted shorts and shirtless for men, fitted shorts and a sports bra for women. Gulick tape body circumferences and skinfold BF% were collected by one of four trained examiners. Interrater reliability of these four examiners was excellent with intraclass correlation coefficient (ICC) = 0.99 (95% CI: 0.99–1.00) based on a single-rating, absolute-agreement, one-way random-effects model.

Conventional body circumferences were measured using Gulick tape (precision: 0.01 cm) at eight anatomical sites. Prior to collecting the measurement, each anatomical site was marked with athletic tape (RockTape, Campbell, CA, USA) to ensure the circumferences were measured at the same location.

- Neck: inferior to the laryngeal prominence
- Waist: most narrow portion between the xiphoid process and umbilicus
- Umbilicus: directly in line with the umbilicus
- Hips: at the widest point and across the most prominent portion of the buttocks
- Left Humerus: midway between the left acromion and olecranon processes
- Right Humerus: midway between the right acromion and olecranon processes
- Left Femur: midway between the left inguinal crease and proximal border of the patella
- Right Femur: midway between the right inguinal crease and proximal border of the patella

Conventional BF% methods included skinfolds and BIA. Skinfolds at nine anatomical sites (chest, abdomen, thigh, bicep, tricep, subscapular, suprailiac, midaxillary, and calf) were collected using a Lange skinfold caliper (Cambridge Scientific Industries, Inc., Cambridge, MD, USA) [28]. Three measurements were collected at each location (precision: 1 mm), averaged for the site, and BF% was calculated using modernized skinfold equations based on sex and race [29,30]. During the BIA measurement, each participant stood upright on the BIA instrument (R20; InBody Co., Seoul, Korea) with the ball and heel of each foot on two metallic footpads while holding a handgrip with both hands pronated and perpendicular to the floor. The participant held the handgrip completely with the palm on one electrode and the thumb resting on the top of the unit’s other electrode. To ensure accuracy, participants adhered to standard pre-measurement BIA guidelines recommended by the American Society of Exercise Physiologists [31].

### 2.3. LeanScreen Methods

For both the LS2D and LS3D exams, each participant stood upright with arms outstretched, motionless, and avoided exaggerated breaths. During the LS2D exam, the participant’s anterior and right lateral photographs were collected using a tablet (iPad 2; Apple Inc., Cupertino, CA, USA). The vertical position of the camera was ensured based on feedback from the LS application. The images were analyzed to determine the count of vertical pixels. A ratio of known linear distance to known pixels was derived from the subject’s known height and known linear distance to the backdrop. In both images, anatomical points were identified for each body area to be measured. Each body area required four points to be identified, two points from each photograph. The four points were used to construct two lines as the major and minor axes of an ellipse, and multiple circular formulas were applied to create an array of estimated circular circumferences. The four points were also used to construct a rectangular plane where additional circular formulas were employed to produce ellipses inscribed within and circumscribed around the rectangular plane. The circumferences of these ellipses were combined into the array of estimated circular circumferences, which was then applied through proprietary algorithms to measure WHR (precision: 0.1) and BF% (precision: 0.1%).

During the LS3D exam, the examiner captured the volume of the participant by maneuvering the same tablet as LS2D equipped with a 3D photonic scanner (Structure Sensor; Occipital, Inc., San Francisco, CA, USA) around the participant. Circumferential measurements of a 3D polygonal mesh model were computed by defining an infinite sized 2D plane to completely intersect the 3D model at a desired location. The intersection provides an array of 3D planar polygonal primitives, triangles, and quadrilaterals as well as the defines 3D vertices of those primitives and the related intersection angles of the 2D plane to each primitive and vertex. The array of primitives was interrogated and the angle of intersection with each primitive applied to calculate the linear distance across the primitive. The summation of all calculated linear distances provided the circumferential measurement of the 3D model at the 2D plane of intersection. The resulting circumferential measurements were then applied through the aforementioned proprietary algorithms to output body circumferences (precision: 1 cm), WHR (precision: 0.01), and BF% (precision: 0.1%).

### 2.4. Data Analysis

We assessed convergent validity and concurrent validity across each pair of proxy methods used to obtain a body composition measure (Table 1). Convergent validity reflects whether two proxy measurements capture the same underlying construct while concurrent validity reflects the correspondence between a proxy measurement and a criterion method when captured simultaneously [32,33].

Convergent validity, an indication that two proxy measurements change proportionally relative to an underlying construct, must be high for the two proxies to provide similar results when used as an outcome in a research study. Based on Carlson and Herdman (2012), convergent validity occurs between two measurements when the lower bound of the 95% confidence interval (CI) of the Pearson correlation coefficient (*r*) is greater than 0.70. On the other hand, concurrent validity is an indication that values obtained from two different proxy measures agree—not only change together as with convergent validity. Concurrent validity was assessed at two levels—across an entire group and for an individual. Group agreement between measurement pairs was assessed using bias and limits of agreement (LoA) [34]. Bias measures how concurrent measurements differ on average across an entire sample, with systematic bias occurring when the 95% confidence interval does not include zero [35]. The 95% LoA indicate the outer extremes for the possible differences between two measurements based on the sample population [36]. Histograms of all differences between measurements were visually inspected for a normal distribution before calculating bias or LoA.

Individual agreement between two measurements was assessed with the percent of participants whose measurements were within the calculated reliable change index (RCI). This clinically useful value indicates when two measures applied to the same person are statistically different from each other [37]. There are multiple methods to set the RCI for comparison, all of which depend on the data available. The RCI for the body circumference comparisons was set using the intraclass correlation coefficient (ICC) method within each comparison [37]. The RCI for all BF% comparisons was performed using the ICC method applied to a comparison of the conventional methods (BIA and skinfolds). This provides a standard comparison on which to assess all other comparisons to conventional methods.

The conventional methods were used as criterion standards for the concurrent validation of LS2D and LS3D. In the analysis of BF%, concurrent validity was assessed relative to the concurrent comparison between the two conventionally collected criterion standards. This enables a fair comparison when evaluating the performance of proxy methods such as LS to measure BF%.

Percent agreement, the proportion of participants who have measures that fall within the bounds of the RCI, were calculated for each concurrent method. For example, an 80% agreement between two concurrent methods means that 8 out of 10 people tested using both methods would have a numerical result that is not statistically significantly different from the other method. All statistical analyses were performed in R (version 3.5.1; R Foundation for Statistical Computing, Vienna, Austria) using packages “psych” and “BlandAltmanLeh” [38,39].

## 3. Results

Almost all measurement pairs across conventional (criterion) and LS methods demonstrated high convergent validity. In the assessment of WHR, LS3D demonstrated high concurrent validity with the Gulick tape method. With regard to BF%, LS2D demonstrated concurrent validity of similar magnitude to the concurrent validity between criterion standards themselves. However, the systematic overestimation of BF% by LS3D relative to criterion standards resulted in lower concurrent validity.

### 3.1. Body Circumferences

When comparing LS3D and Gulick tape methods for measuring body circumferences (Table 2), convergent validity was excellent. Group biases were generally small with some large limits of agreement corresponding to those with large bias. Individual agreement between the methods was poor for most locations. Of note, there were several output errors by LS3D in the assessment of both right and left humerus circumferences that resulted in nonviable, non-geometric measurements. This tended to occur in very thin subjects, which our sample was biased towards given the population from which they were sampled. As a result, the sample size is reduced for humerus circumference comparison between LS3D and Gulick tape.

The minimum criterion for convergent validity between Gulick tape and LS3D was met for each body circumference location. Even the most conservative estimate of the correlation coefficient (lower bound of the 95% CI) met or exceeded 0.8 at all 8 sites, and convergent validity for the waist was very high (*r* > 0.94).

There was no statistically significant group bias in 4 of the 8 body circumference locations (Neck, Waist, Left Humerus, Right Humerus). Group bias was statistically different from zero between Gulick tape and LS3D for 4 of 8 locations (Umbilicus, Hip, Left Femur, and Right Femur). The LS3D bias was 2 cm higher than Gulick tape in each location with statistically significant bias. The largest limits of agreement occurred at the Umbilicus and Hip—which also had significant biases—with upper limits of 8 cm higher as measured by LS3D relative to Gulick tape.

**Figure 1:**
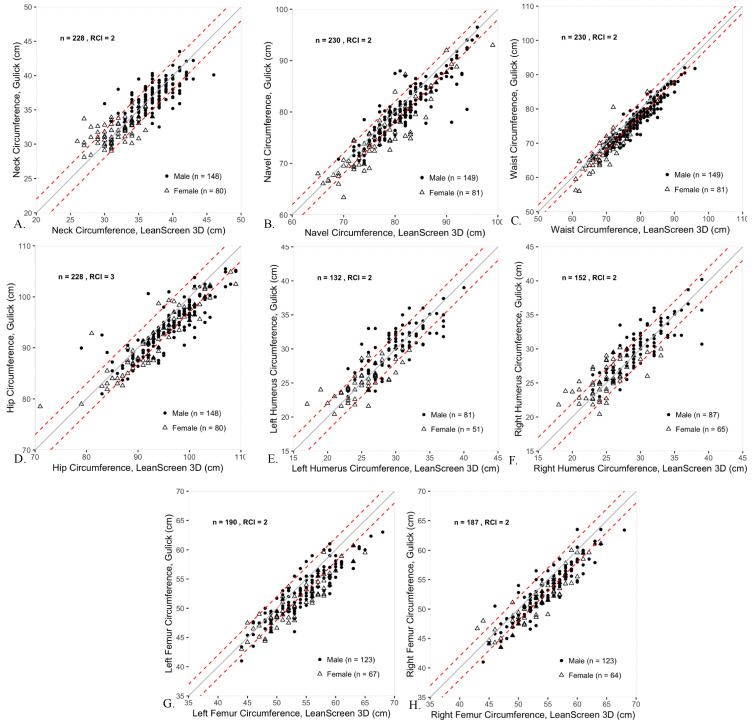
Scatter plots comparing circumferences at the 8 different anatomical sites as measured by LS3D (*x*-axis) and Gulick tape (*y*-axis). The dashed lines represent the RCIs, which are 2 cm for all measures except at the hip (RCI = 3 cm). The solid line is the unity line. Males are represented as filled circles, while females are represented as open triangles.

Individual agreement between LS3D and Gulick tape was moderate for the neck, in which 75% of the participants were within the RCI of 2 cm (Table 2, Figure 1). However, individual agreement between LS3D and Gulick tape was low for the 7 other locations, ranging from 46 to 69% agreement within 2 cm of Gulick Tape (Table 2). RCIs were 2 cm for all locations except the Hip, which had an RCI of 3 cm (Figure 1).

### 3.2. Waist-To-Hip Ratio

**Figure 2:**
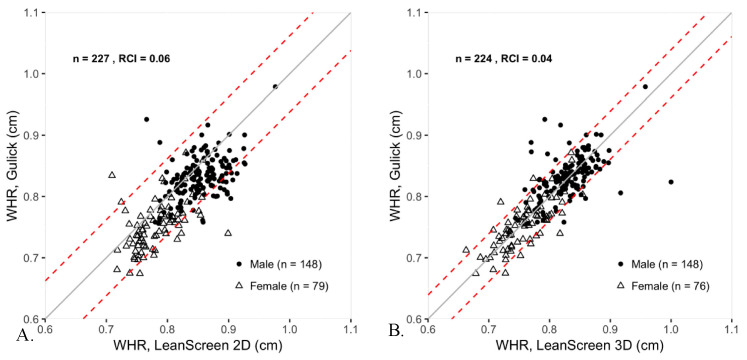
Scatter plots comparing WHR as measured by LS2D, LS3D, and Gulick tape. The dashed lines represent the RCIs, which are 0.06 for LS2D and 0.04 for LS3D. The solid line is the unity line. Males are represented as filled circles, while females are represented as open triangles.

In the determination of WHR, LS3D outperformed LS2D and demonstrated high convergent and concurrent validity with Gulick tape. LS2D did not meet the 0.7 threshold for convergent validity if using the conservative lower bound of the 95% CI of the Pearson coefficient, and had a statistically significant group bias of 0.02 with LoA ranging from −0.05 to 0.10 (Table 3). Individual agreement between LS2D and Gulick tape was high with 85% of participants falling within the RCI of 0.06 (Figure 2). In contrast, LS3D showed high convergent validity, very little bias, and narrow limits of agreement ranging from −0.06 to 0.06 (Table 3). Individual agreement between LS3D and Gulick tape was also high with 87.1% of participants falling within the RCI of 0.04 (Figure 2).

### 3.3. Body Fat Percentage

**Figure 3:**
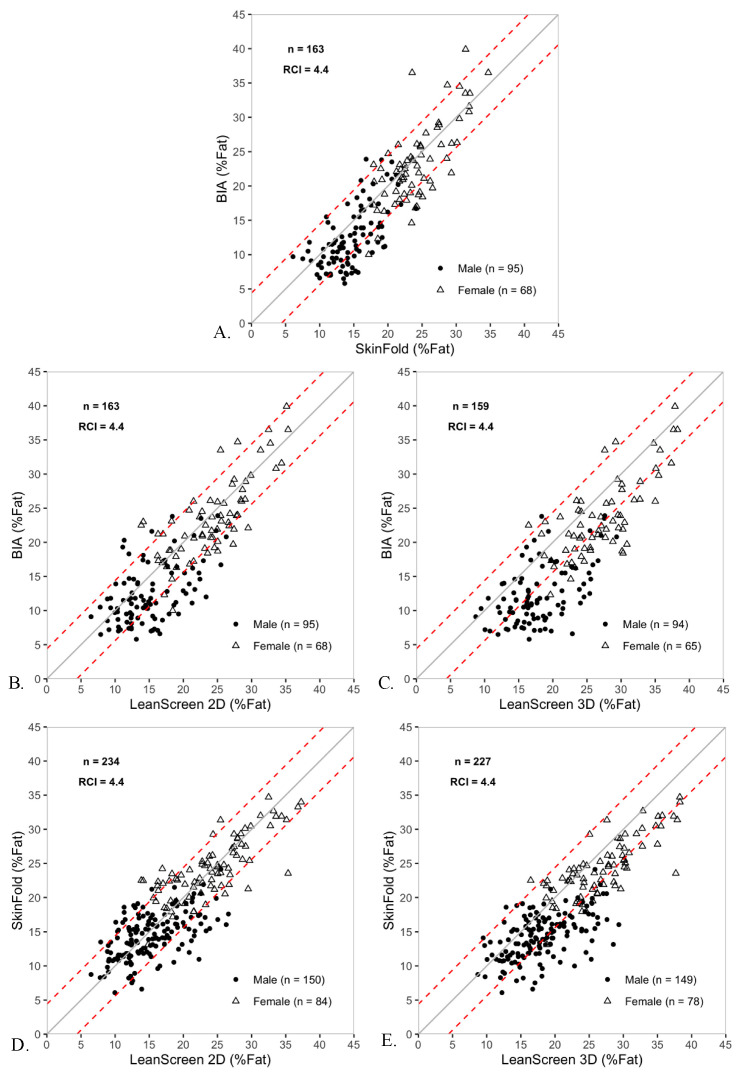
Scatter plots comparing BF% as measured by LS2D, LS3D, skinfolds, and BIA. The dashed lines represent the RCI which was 4.4% for all comparisons. The solid line is the unity line. Males are represented as filled circles, while females are represented as open triangles.

Convergent validity was acceptable for all comparisons between methods. Concurrent validity for the two conventional standards showed a small group bias with wide LoA and individual agreement in approximately 7 out of 10 participants. Concurrent validity for LS2D and the two conventional methods was equivalent or exceeded those in the conventional comparison. Concurrent validity for LS3D was lower than the conventional comparison as a result of a consistent overestimation of BF%. Of note, due to equipment malfunction in the late phase of data collection, fewer subjects were assessed with BIA than with skinfolds—accounting for the sample size discrepancy observed in Figure 3.

For the two criterion standard methods of BF%—skinfolds and BIA—there was high convergent validity (0.82) and a small, but statistically significant, bias of 1.7% for the group (Table 4). LoA between skinfolds and BIA spanned 14.7% (−5.7 to 9.0) for the group. The RCI based on ICCs between the conventional methods was 4.4% and set as the standard for all subsequent comparisons between LS and conventional methods. Individual agreement between the conventional methods indicated that 68.1% (approximately 7 out of 10) participants would not measure differently between methods, which corresponds to the significant bias observed. (Figure 3).

In the comparison of BF% as determined by BIA and LS2D, the threshold for convergent validity was met (0.77) and a small, but statistically significant, bias of 1.8% (LS2D overestimated relative to BIA) existed for the group (Table 4). LoA between BIA to LS2D spanned 16.6% (−6.5, 10.1) for the group. Individual agreement between the BIA and LS2D indicated that 65.6% (approximately 7 out of 10) participants would not measure differently between methods, which corresponds to the significant bias observed (Figure 3). Group bias, span of LoA, and individual agreement between BIA and LS2D were all similar to the concurrent validity between the criteria standards.

In the comparison of BF% as determined by skinfolds and LS2D, the threshold for convergent validity was met (0.78) and no significant bias existed in the group (Table 4). LoA between skinfolds to LS2D spanned 14.6% (−6.8, 7.8) for the group. Individual agreement between skinfolds and LS2D indicated that 77.4% (approximately 8 out of 10) participants would not measure differently between methods, which corresponds to the absence of significant bias observed (Figure 3). Group bias, span of LoA, and individual agreement between skinfolds and LS2D were all smaller than the same measures of concurrent validity between conventional methods.

In the comparison of BF% as determined by BIA and LS3D, the threshold for convergent validity was met (0.76) and a statistically significant bias of 4.8% (LS3D overestimated relative to BIA) existed for the group (Table 4). LoA between BIA to LS3D spanned 17.0% (−3.7, 13.3) for the group. Individual agreement between the BIA and LS3D indicated that 42.1% (approximately 4 out of 10) participants would not measure differently between methods, which corresponds to the significant bias in the group (Figure 3). Compared to the measures of concurrent validity between conventional methods (skinfolds and BIA), group bias was three times larger, LoA were wider, and individual agreement lower between BIA and LS3D.

In the comparison of BF% as determined by skinfolds and LS3D, the threshold for convergent validity was met (0.77) and a statistically significant bias of 3.4% (LS3D overestimated relative to skinfolds) existed for the group (Table 4). LoA between skinfolds to LS3D spanned 14.9% (−4.1, 10.8) for the group. Individual agreement between the skinfolds and LS3D indicated that 61.7% (approximately 6 out of 10) participants would not measure differently between methods, which corresponds to the significant bias in the group (Figure 3). Compared to the measures of concurrent validity between conventional methods, group bias was two times larger, LoA were slightly wider, and individual agreement lower between skinfolds and LS3D.

## 4. Discussion

This investigation examined the validity of a novel 2D digital photography (LS2D) and 3D photonic scanning (LS3D) software compared to conventional criterion methods of determining body composition. The RCI based on the conventional methods comparison was set as an objective maximum threshold of agreement for all BF% comparisons. In the determination BF%, LS2D exhibited higher agreement than LS3D with BIA and skinfold methods. However, a comparison with reference literature suggests LS3D has high agreement with DXA [40]. In the assessment of WHR, LS3D demonstrated high agreement relative to Gulick tape despite poor agreement on absolute waist and hip circumferences. We recommend choosing either LS2D or LS3D as a proxy for BF% based on the criterion desired by the user. Future investigations comparing LS3D to DXA are needed to confirm the potential agreement between these methods.

Our validity measures are congruent with existing literature comparing conventional methods used to estimate BF%. In two comparisons to DXA—the current gold standard—Chen et al. reported that BIA underestimated DXA by −3.7% with an LoA that spanned 16.4% (n = 711) and MacDonald et al. reported that the Department of Defense equations underestimate DXA by 4.8% with an LoA that spanned 13.5% (n = 148) [27,40]. In our comparison between conventional methods, BIA underestimated skinfolds by 1.7% with LoA spanning 14.6% (n = 163). In our various comparisons between LS and conventional methods, biases ranged from 0.5% (LS2D vs. skinfolds) to underestimating by 4.8% (LS3D vs. BIA) and LoA ranged from 14.6 to 17.0%. It is notable that the reported span of the LoA for BF% is up to 16.4% between conventionally accepted methods. Pearson correlation coefficients were comparable to the values of 0.82–0.83 reported in Marx et al. [41]. To our knowledge, no comparison for individual agreement is available, so we will reference the 7 out of 10 participant agreement between our conventional methods, BIA and skinfolds. WHR outcomes are also in a comparable range to previous work by Marx et al. [41].

The suitability of LS2D and LS3D to replace conventional BF% methods must be guided by the user’s preferred method of measurement. LS2D exhibited higher concurrent validity with the skinfolds and BIA methods than LS3D. As such, if the user prefers skinfolds as their standard, LS2D would be an appropriate replacement due to its lack of bias, similar LoA, and high individual agreement (8 of 10 participants agreeing). Similarly, if the user prefers BIA, LS2D would be an appropriate replacement due to its lower bias and comparable LoA relative to other comparisons of conventional methods (BIA vs. DXA, BIA vs. skinfolds). If absolute agreement is not required, the high convergent validity (>0.82) indicates that LS2D is able to reliably detect a change in BF% with either skinfolds or BIA as the standard method of measurement. LS2D overestimated BIA in the current study (1.8%) but was reported as underestimating DXA (3.3%) by Chen et al., suggesting that LS2D may approximate DXA better than BIA. However, the direct comparison between LS2D and DXA reported in MacDonald et al. indicates a similar agreement of BIA and LS2D with DXA.

In the measurement of BF%, LS3D exhibited lower concurrent validity than LS2D when compared to conventional methods. The overestimation of LS3D by 4.8% (relative to BIA) and 3.4% (relative to skinfolds), as well as the poor individual agreement (4 of 10 participants relative to BIA and 6 of 10 participants relative to skinfolds) indicates that LS3D is a poor replacement for these criterion measures when absolute agreement with BF% measures is required by the user. However, LS3D would be an appropriate replacement to determine group means in research and reliably detecting a change in BF%, given its strong convergent validity (>0.76 and >0.77) and comparable LoA with both BIA and skinfold methods (Carlson and Herdman, 2012) [32].

**Figure 4:**
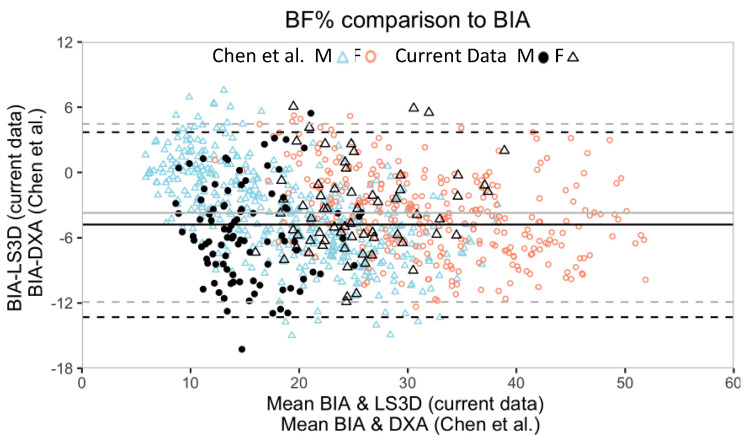
Bland-Altman plots comparing BF% as measured by BIA and LS3D (current study) and overlayed with BIA and DXA (Chen et al.) [40]. Both studies, which are well powered, show a similar bias of DXA and LS3D to BIA and similar span of LoA. Color and formatting in each study are preserved with lighter colors representing Chen et al.

When evaluating our results in the context of a well-powered comparison of BIA and DXA, circumstantial evidence suggests LS3D may approximate DXA. Chen et al. compared BF% measured by a similar 8 electrode BIA (Tanita BC-418) and DXA in a sample of 711 Chinese participants [40]. When our data are superimposed with Chen et al. (Figure 4), their similarities become apparent—LS3D and DXA both measure higher values than BIA with a similar bias (3.7% vs. 4.8%) and comparable span of LoA (16.4% and 17.0%). The inferred bias of LS3D and DXA is approximately 1%, which indicates that, although LS3D does not agree well with BIA or skinfolds, it may have high agreement with DXA. This assumes that the BIA devices in each study perform similarly in the populations tested. Both were 8-electrode devices, which allows a multi-segmental approach to measuring fat in the human body based on five heterogeneous cylinders with separately measured resistances. We previously demonstrated the superior reliability of an 8-electrode BIA device compared to a 4-electrode device [42]. Future work assessing the accuracy of LS3D should include a comparison to DXA, which is considered most accurate.

In contrast to concurrent validity, convergent validity was consistently high across all comparisons in this study excluding WHR as measured by LS2D and Gulick tape. This is despite use of the most conservative estimate—the lower bound of the 95% confidence interval for the Pearson Coefficient. High convergent validity indicates that almost all proxy method pairs were indeed measuring the same underlying construct. In practice, this means these methods vary proportionally with one another and can reliably detect changes.

In the measurement of WHR, a clinically useful body composition measure, LS3D performed much better than LS2D when compared to the Gulick tape. LS3D exhibited no bias, a narrow LoA (−0.06 to 0.06), and high individual agreement (9 out of 10 participants agreeing). Notably, the high WHR validity occurred despite LS3D having low agreement with the Gulick tape on absolute waist and hip circumference measurements, indicating high proportional accuracy between measurement sites. In contrast, LS2D demonstrated a small but statistically significant overestimation of WHR—albeit with high individual agreement (85%). In practice, for the acquisition of WHR, LS3D is a reasonable substitute for Gulick tape despite low concurrent validity on the circumference measurements individually. This is compounded by the practical ease of using a 3D scanner compared to the often-cumbersome nature of a physical tape measure.

Interestingly, our results indicate a gender discrepancy in the determination of BF%. Across all method pair comparisons in the assessment of concurrent validity, female subjects demonstrated higher individual agreement (Table 4). However, this gender effect was largely attenuated in the assessment of circumferences (Table 2) and reversed in the assessment of WHR (Table 3). One possible etiology of this discrepancy might be differences in clothing worn—males wore only fitted shorts while females wore fitted shorts and a sports bra. Further investigation into gender discrepancies in digital anthropometry may be warranted.

We have attempted to provide a thorough interpretation of convergent and concurrent validity that extends beyond group inference expectations for individual measurements when using LS as a proxy measure for body composition. Although the correlation coefficient is an excellent indicator of the convergent form of construct validity for an entire group of measurements, it does not provide information about agreement and concurrent validity for a group or individual measurement [35]. Similarly, bias (mean difference) provides information about how a given method will perform across a sample of similar size and characteristics and, if consistent, the proxy method can be adjusted by subtracting the mean difference from the proxy measurements [43].

Although more applicable than bias for understanding individual measurements, the LoA, when reported, are not interpreted in the context of standard measurement comparisons. The LoA are an estimate of the “extreme values” in which the nearly all difference pairs will fall [36]. In a previous comparison using LS2D the “wide LoA” of 14.0% was cited as one of the reasons for not recommending its use as a proxy for BF% [27]. However, we found that the span of the LoA in MacDonald et al., this study, and others using LS were the same or sometimes even lower than the LoA between conventional measures of BF%.

To compensate for the lack of inference on individuals, we calculated the percent agreement based on thresholds of the RCI [37]. This approach determines whether a data point agrees or disagrees based on an a priori maximum acceptable threshold and results that can be conveyed in clinically useful language such as “8 out of 10 participants will measure the same as BIA method” [35]. We set the threshold for agreement in this study to 4.4 BF%, which is the RCI calculated using the ICC method between BIA and skinfolds, two accepted methods [37]. Indexing the RCI to a comparison between two conventional methods provided a common standard to be applied for all BF% comparisons. Our group and MacDonald et al. used similar methods with a priori thresholds set as 3 BF% and 4 BF%, respectively [27,42]. These thresholds, which were more strict than the conventional methods could perform in our analysis, seem to be aspirational for all proxy BF% calculations.

The most apparent limitation of this study was the lack of DXA as a criterion comparison in the assessment of BF%. DXA is widely considered the gold standard for assessing BF%, though it is not without drawbacks—namely its cost and scarcity. While DXA was not feasible in this study, we previously demonstrated concurrent validity of the R20 BIA instrument when compared to DXA, albeit in a small sample size of convenience [42]. We chose BIA and skinfolds as conventional methods in this study due to their ubiquity in use—thus making our results more relevant to the typical user. Future studies should investigate the convergent and concurrent validity between LS technology and DXA directly.

## 5. Conclusions

Despite tremendous advancement in digital anthropometry over the past decade, the field remains in its infancy. Yet its potential as a viable solution to the cumbersome nature of traditional measurement methods should not be underestimated, especially in the context of personal fitness and health monitoring. In most cases, there exists a trade-off between accuracy and practicality across anthropometric tools.

In the current study, we demonstrate that LS2D has high convergent and concurrent validity in BF% when compared to BIA and skinfolds, while LS3D has lower measures of concurrent validity. In comparison with another large study, LS3D may agree well with DXA, but needs further verification in a well-designed study. In contrast, LS3D exhibited high concurrent validity with Gulick tape in the assessment of WHR, while LS2D exhibited lower concurrent validity. Because gold-standard instrumentation is unlikely to become more compact, simplistic, and affordable for everyday use, efforts must be aimed at increasing the validity of existing modalities such as digital photography and photonic scanning.

## Figures and Tables

**Table 1:** List of conventional and LeanScreen methods used for determining each measure of body composition.

Body Composition Measure	Conventional Methods	LeanScreen Methods
Body Circumferences	Gulick tape	LeanScreen 3D (LS3D)
Waist-To-Hip Ratio (WHR)	Gulick tape	LeanScreen 2D (LS2D) LeanScreen 3D (LS3D)
Body Fat Percentage (BF%)	SkinfoldsBioelectrical Impedance (BIA)	LeanScreen 2D (LS2D) LeanScreen 3D (LS3D)

**Table 2:** Validity measures of body circumferences as measured by Gulick tape and LS3D.

Body Circumference Locations	Convergent Validity	Concurrent Validity				
	Pearson Coefficient *r* [95% CI]	Bias [95% CI]	95% Limits of Agreement	Individual % Agreement **		
				Male	Female	Total
**Neck (cm)**	0.85 [0.80, 0.88]	0 [−1, 1]	(−4, 4)	74 (4)	78 (5)	75 (3)
**Umbilicus (cm)**	0.91 [0.89, 0.93]	2 [1, 3] *	(−4, 8)	62 (4)	51 (6)	58 (3)
**Waist (cm)**	0.96 [0.94, 0.97]	2 [−6, 6]	(−2, 6)	46 (4)	46 (6)	46 (3)
**Hip (cm)**	0.89 [0.86, 0.91]	2 [1, 3] *	(−4, 8)	69 (4)	70 (5)	69 (3)
**Left Humerus (cm)**	0.85 [0.80, 0.89]	0 [−1, 1]	(−4, 4)	62 (5)	76 (6)	67 (4)
**Right Humerus (cm)**	0.85 [0.80, 0.89]	0 [−1, 1]	(−4, 4)	67 (5)	69 (6)	68 (4)
**Left Femur (cm)**	0.90 [0.87, 0.92]	2 [1, 3] *	(−2, 6)	53 (5)	42 (6)	49 (4)
**Right Femur (cm)**	0.92 [0.89, 0.94]	2 [1, 3] *	(−2, 5)	56 (4)	50 (6)	54 (4)

*—indicates statistically significant bias between the measurement methods. **—RCI = 2 cm for all measures except Hip, which has RCI = 3 cm.

**Table 3:** Validity measures of WHR as measured by Gulick tape, LS2D, and LS3D.

Waist-to-Hip Ratio Measurements	Convergent Validity	Concurrent Validity				
	Pearson Coefficient *r* [95% CI]	Bias [95% CI]	95% Limits of Agreement	Individual % Agreement **		
				Male	Female	Total
**Gulick and LeanScreen 2D**	0.73 [0.66, 0.78]	0.02 [0.01, 0.03] *	(−0.05, 0.10)	86.5 (2.8)	82.3 (4.3)	85.0 (2.4)
**Gulick and LeanScreen 3D**	0.81 [0.75, 0.85]	0.00 [−0.01, 0.01]	(−0.06, 0.06)	87.8 (2.7)	85.5 (4.0)	87.1 (2.2)

*—indicates statistically significant bias between the measurement methods. **—RCIs were 0.06 for LS2D and 0.04 for LS3D.

**Table 4:** Validity measures of body fat percentage as determined by LS2D, LS3D, skinfolds, and BIA.

BF% Measurements	Convergent Validity	Concurrent Validity				
	Pearson Coefficient *r* [95% CI]	Bias [95% CI]	(95% LoA)	Individual % Agreement (SD) **		
				Male	Female	Overall
**BIA and Skinfolds**	0.86 [0.82, 0.90]	1.7 [1.1, 2.3] *	(−5.7, 9.0)	65.3 (4.9)	72.1 (5.4)	68.1 (3.7)
**BIA and LeanScreen2D**	0.82 [0.77, 0.87]	1.8 [1.2, 2.4] *	(−6.5, 10.1)	58.9 (5.0)	75.0 (5.3)	65.6 (3.7)
**Skinfolds and LeanScreen2D**	0.83 [0.78, 0.86]	0.5 [0.0, 1.0]	(−6.8, 7.8)	74.7 (3.6)	82.1 (4.2)	77.4 (2.7)
**BIA and LeanScreen3D**	0.82 [0.76, 0.86]	4.8 [4.1, 5.5] *	(−3.7, 13.3)	38.3 (5.0)	47.7 (6.2)	42.1 (3.9)
**Skinfolds and LeanScreen3D**	0.82 [0.77, 0.86]	3.4 [2.9, 3.9] *	(−4.1, 10.8)	57.7 (4.0)	69.2 (5.2)	61.7 (3.2)

*—indicates statistically significant bias between the measurement methods. **—RCI = 4.4% for all measures.
